# ESR Bridges: imaging and treatment in brain tumours—a multidisciplinary view

**DOI:** 10.1007/s00330-024-11279-1

**Published:** 2024-12-19

**Authors:** Marion Smits, Niels Verburg, Martin J. van den Bent

**Affiliations:** 1 Department of Radiology & Nuclear Medicine, Erasmus MC, Rotterdam, The Netherlands; 2 Brain Tumour Centre, Erasmus MC Cancer Institute, Rotterdam, The Netherlands; 3 Medical Delta, Delft, The Netherlands; 4 Department of Neurosurgery, Amsterdam UMC, Amsterdam, The Netherlands; 5 Cancer Centre Amsterdam, Amsterdam UMC, Amsterdam, The Netherlands

## What is the clinical need to be addressed?

Primary brain tumours are among the most lethal types of cancer, with a 5-year survival of the most malignant tumour (glioblastoma) of only five percent [1]. Diffuse gliomas are the most common primary brain tumours, with an incidence of 6/100,000 worldwide. They arise from the oligodendrocytes or astrocytes that support the neurons in the brain. Their growth pattern is diffuse, resulting in a widespread infiltration of the brain at the time of diagnosis, greatly hampering effective treatment. Diffuse gliomas can be divided into two molecular groups based on the presence or absence of mutation in the isocitrate dehydrogenase (IDH) 1 or 2 genes: IDH-mutant and IDH-wildtype, respectively. IDH-mutant diffuse glioma occurs more commonly in younger patients and can evolve from a lower to a higher, more aggressive grade, while IDH-wildtype diffuse glioma occurs mostly in older patients and starts at the highest malignancy grade [2]. The primary treatment of all diffuse gliomas is maximal safe resection [3]. In low-grade glioma (LGG), this is followed by either radiological follow-up or chemoradiation in case of a higher risk of recurrence. In the near future, the IDH inhibitor vorasidenib is likely to play a role as well [4]. In high-grade glioma (HGG), resection is always followed by chemoradiation. In the case of non-resectable tumours, a biopsy is performed to obtain tissue for diagnosis.

Imaging is indispensable for the diagnosis, treatment planning and follow-up of diffuse gliomas, but several challenges remain to be addressed.

## Challenges and opportunities—radiologist’s view

MRI is the imaging modality of choice for differential diagnosis and tumour characterisation. Diagnostic challenges remain for differentiation between diffuse glioma and other neoplastic lesions. In presumed glioma, both IDH-mutational status and grade are relevant prior to surgery as both determine prognosis and surgical approach [3]. Traditionally, contrast-enhancement has been considered an indication of high grade, but about 25% of grade 2 LGG are known to enhance, while up to 30% of non-enhancing glioma were found to be high grade [5]. Advanced MRI techniques, in particular perfusion-weighted imaging and MR spectroscopy, are more accurate in determining biological aggressiveness, both in terms of molecular type and tumour grade [6].

For response assessment, two main challenges can be identified. The first concerns the accurate assessment of response to treatment in terms of tumour size. The response assessment criteria for neuro-oncology (RANO) prescribe in their latest update (2.0) bidimensional size assessment with the option to replace these with volumetric measurements [7]. In particular, in irregular, slow-growing LGG, volumetric assessment is thought to be more sensitive to determine treatment response or tumour progression. The lack of robust automated assessment tools, however, hinders volumetric assessment within a clinical workflow. The second challenge concerns the differentiation between tumour progression and treatment-related radiological abnormalities, which are indistinguishable on conventional MRI. Both advanced MRI and positron emission tomography (PET) may aid in their distinction, but as yet, no fully accurate imaging technique is available.

## Challenges and opportunities—other clinician’s view

Local treatment is currently guided by conventional MRI, which provides limited information on the tumour load in both LGG and HGG. Traditionally, in presumed HGG, the contrast-enhancing portion is targeted, while in presumed LGG, the entire region of T2-weighted hyperintensity is considered to be a tumour. Both estimates are known to be incorrect: tumour cells are present outside the region of enhancing and even non-enhancing signal abnormalities, while the T2w-hyperintense region consists of a mixture of tumour and oedema. Accurate tumour delineation represents a major challenge with implications for the resection and the radiotherapy volumes. Recent studies found a survival benefit of resections extending beyond standard imaging in both LGG and HGG [8, 9]. There is thus a clear need for imaging that could better guide local treatment beyond conventional MRI abnormalities. Advanced MRI techniques and PET are known to be more accurate than conventional MRI for the detection of tumour presence in HGG. Their uptake in clinical treatment guidelines is essential but requires high quality, prospective studies to provide the necessary evidence for their added value [10].

A further clinical challenge is the fact that current treatment decision-making generally relies on qualitative assessment. Quantitative assessment of tumour burden and analyses taking physiological information from advanced MRI and/or PET into account could help to improve treatment decision-making by improved awareness of tumour volume and aggressiveness.

## Recommendations for clinical practice

- The RANO criteria are based on conventional MRI and rely primarily on 2D measurement of contrast enhancement. Further improvement is needed in the context of current and upcoming systemic treatments.
- To delineate tumour and tumour infiltration conventional MRI is insufficient, and both advanced MRI and PET should be taken into account in future guidelines for local treatment planning.
- Insights into differences in tumour behaviour based on molecular profiles have sparked the debate about the extent of tumour resection
  - Should we extend beyond contrast-enhancement for IDH-wildtype glioblastoma?
  - Should we extend beyond T2w-/T2w-FLAIR abnormalities for IDH-mutant LGG?
    Assuming that this is safely possible in a particular patient.

## Future direction

It is clear that advanced imaging techniques are essential to guide future developments in glioma diagnosis, treatment planning and treatment monitoring. Additionally, artificial intelligence algorithms are being developed to improve initial diagnosis, assess tumour volumes and other markers of response to treatment, and predict outcome using a combination of imaging and non-imaging parameters. Prediction of molecular tumour types prior to surgery using advanced neuroimaging non-invasively (a so-called ‘virtual biopsy’) allows better-informed decision-making for both patients and healthcare professionals.

A more personalised resection based on molecular profile and biological/advanced imaging will allow a risk adapted strategy for safe resection. Along similar lines, radiation therapy can be personalised by the of use biological target volumes based on advanced neuroimaging, limiting exposure to healthy brain tissue and thus reducing—cognitive—side effects.

Treatment monitoring with advanced imaging will allow the identification of early responders and the distinction between tumour progression from pseudoprogression, allowing the selection of the right approach in individual patients.

Quantification plays a key role, both in terms of quantitative imaging markers and for measuring tumour burden. With quantitative imaging markers, specific tumour characteristics can be compared between and within patients, which is essential for both clinical practice and research. This will deepen our understanding of disease processes and treatment responses. Insight into the tumour microenvironment, the blood-brain barrier, and the expression of targetable receptors provided by advanced neuroimaging can boost drug development.

For all this, it is adamant that advanced imaging techniques are incorporated into clinical practice with clear guidance on their optimal implementation and interpretation and with a solid scientific foundation.
